# Risk factors for post-traumatic lymphedema after open fractures of the lower leg: a retrospective cohort study

**DOI:** 10.3389/fsurg.2026.1931485

**Published:** 2026-09-11

**Authors:** Xiao Huang, Yuji Zhang, Zhenkun Lv, Zhijin Liu, Shuai Dong, Ruowen Zhou, Wen Ju, Jihui Ju, Ruixing Hou

**Affiliations:** 1 Department of Joint Surgery, Suzhou Ruihua Orthopedic Hospital, Suzhou, Jiangsu, China; 2 Suzhou Medical College of Soochow University, Suzhou, Jiangsu, China

**Keywords:** free flap, lower leg, open fracture, post-traumatic lymphedema, risk factors

## Abstract

**Background:**

Post-traumatic lymphedema (PTL) is an underrecognized complication of severe limb trauma and is characterized by persistent limb swelling. Patients with open fractures of the lower leg may be more susceptible to PTL, and clinical factors such as extensive soft-tissue injury, wound infection, and repeated surgical procedures may be associated with lymphatic injury and local inflammation. However, the clinical factors associated with PTL in this population remain unclear. This study aimed to identify factors associated with PTL after open fractures of the lower leg.

**Methods:**

We retrospectively screened 129 patients treated for open fractures of the lower leg between March 2023 and March 2025. After excluding one patient who underwent amputation and seven who were lost to follow-up, 121 patients were included and assessed for post-traumatic lymphedema at 6 months after the initial operation. Binary logistic regression was used to identify associated factors.

**Results:**

Of the 121 patients, 41 (33.88%) developed PTL. Multivariable logistic regression showed that increasing age (odds ratio [OR] = 1.05, 95% confidence interval [CI] 1.02–1.09; *P* = 0.002), postoperative infection (OR = 5.15, 95% CI 1.25–21.19; *P* = 0.023), and skin and soft-tissue necrosis-related defects (OR = 5.76, 95% CI 2.04–16.31; *P* < 0.001) were associated with PTL. Diabetes mellitus did not reach statistical significance, although the point estimate was elevated (OR = 8.00, 95% CI 0.77–82.97; *P* = 0.081). In the extended sensitivity model, increasing age and postoperative infection remained associated with PTL, whereas the associations of the two injury-severity indicators were attenuated. In the alternative injury-severity model, Gustilo–Anderson type IIIB/IIIC injury was associated with PTL.

**Conclusions:**

Increasing age and postoperative infection were consistently associated with PTL after open fractures of the lower leg. Skin and soft-tissue necrosis-related defects and Gustilo–Anderson type IIIB/IIIC injuries showed model-dependent associations. These findings may support targeted postoperative surveillance.

## 1 Introduction

**Figure 1:**
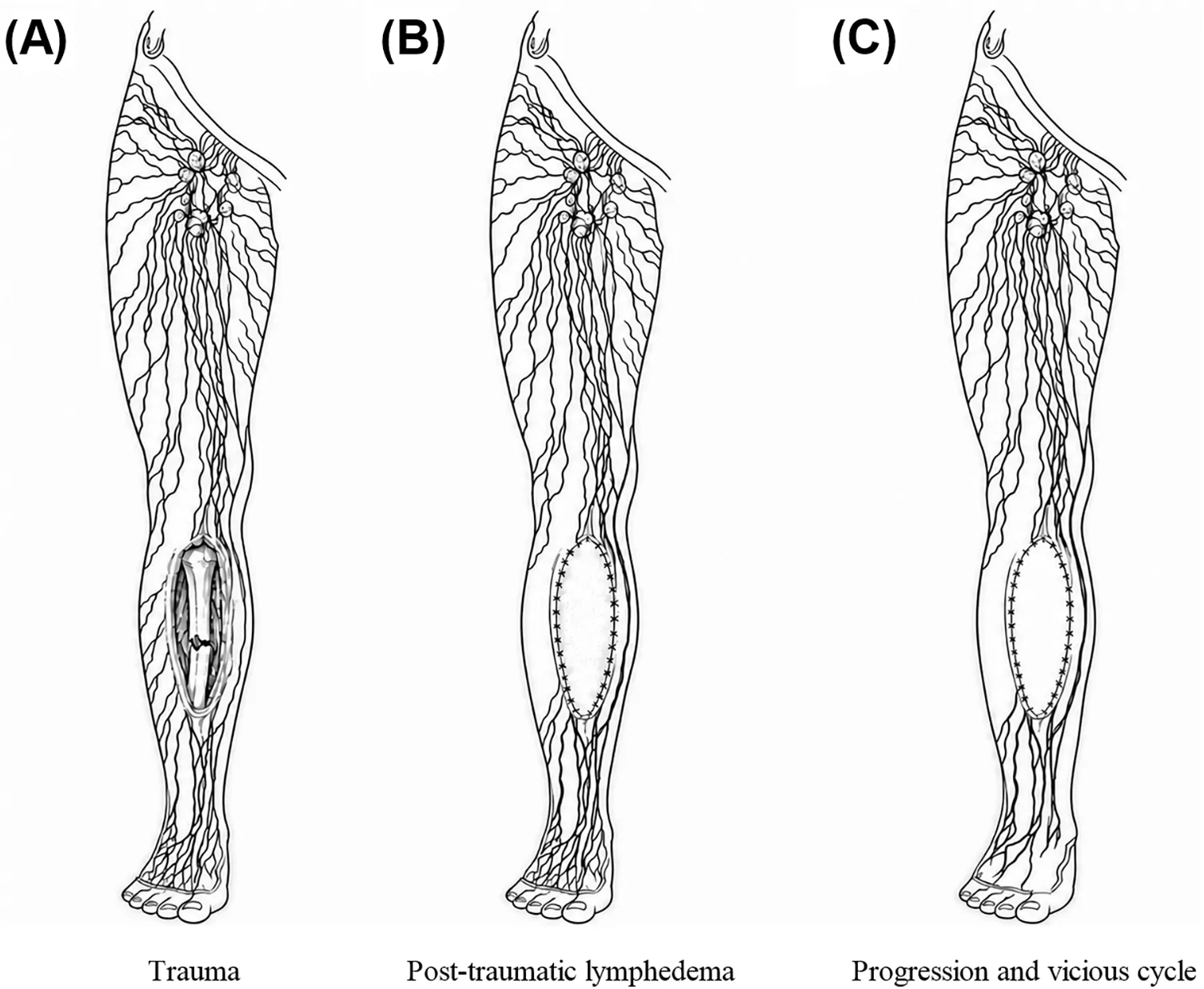
Conceptual model of the progression from traumatic lymphatic injury to post-traumatic lymphedema (PTL). **(A)** Trauma: Mechanical injury causes lymphatic vessel disruption or defects. Lymphatic fluid is rerouted through the remaining lymphatic pathways, while reduced lymphatic transport efficiency contributes to the development of early post-traumatic edema. **(B)** PTL: Restoration of lymphatic drainage pathways is limited, and the reconstructed soft tissue lacks functional lymphatic vessels, while persistent inflammation increases microvascular permeability and tissue-fluid production. Although the remaining lymphatic vessels undergo compensatory dilation, this response is insufficient to meet the increasing drainage demand, resulting in persistent swelling of the affected limb. **(C)** Progression and vicious cycle: As lymphatic compensatory function further declines, lymphatic stasis progressively worsens, and tissue fibrosis develops. Persistent lymphatic dysfunction increases susceptibility to infection, which may further impair lymphatic drainage, ultimately establishing a vicious cycle of edema, infection, and worsening edema. Source: Adapted from Bi YS (27), © Editorial Office of Journal of Shandong University (Health Sciences), with permission.

Post-traumatic lymphedema (PTL) remains an underrecognized complication following severe limb trauma. It is characterized primarily by persistent swelling of the affected limb and may be accompanied by tightness, pain, limb heaviness, functional impairment, and reduced quality of life (1, 2). Unlike transient postoperative edema, PTL is essentially a form of secondary chronic lymphedema (1, 3). During the early phase after severe trauma, mechanical injury may disrupt or destroy lymphatic vessels, resulting in impaired lymphatic drainage (4, 5). Before lymphatic drainage pathways are restored, lymphatic fluid may be rerouted through the remaining lymphatic channels, while the residual lymphatic vessels undergo compensatory dilation to maintain a certain degree of drainage capacity (6–8). However, persistent local inflammation increases microvascular permeability and tissue-fluid production, thereby further increasing the drainage burden on the lymphatic system (4, 5, 9). When lymphatic regeneration is impaired and compensatory dilation is insufficient to meet the progressively increasing drainage demand, lymphatic stasis gradually worsens and ultimately leads to PTL. Prolonged lymphatic stasis may further drive the dilated lymphatic vessels from a compensatory to a decompensated state, accompanied by tissue fibrosis and irreversible lymphatic dysfunction (10, 11). It may also impair local tissue-fluid clearance and immune-cell trafficking, thereby increasing susceptibility to erysipelas, cellulitis, and other skin and soft-tissue infections (11, 12). Recurrent infection may, in turn, further damage the lymphatic vessels, creating a self-perpetuating cycle of lymphatic dysfunction and inflammation (4, 5, 11). This proposed pathophysiological process is illustrated in the conceptual model shown in Figure 1.

Open fractures of the lower leg are frequently accompanied by severe soft-tissue injury, wound contamination, and vascular damage, and patients often require repeated debridement and reconstructive procedures. The initial trauma, secondary infection, tissue necrosis, and subsequent surgical interventions may all disrupt the superficial lymphatic vessels and trigger persistent local inflammation. Wagner et al. (13) reported that symptoms suggestive of secondary lymphedema were common among patients with lower-extremity open fractures accompanied by severe soft-tissue injury. Mello et al. (14) demonstrated marked impairment of lymphatic circulation following circumferential degloving injuries of the lower extremities. Van Zanten et al. (15) further observed that, after free-flap reconstruction for high-energy open tibial fractures, scar tissue at the recipient-site margin may obstruct lymphatic drainage, forcing lymphatic flow to bypass the injured region and thereby reducing lymphatic transport efficiency, which may contribute to the development and progression of PTL. Given that conventional flap reconstruction does not necessarily restore disrupted lymphatic drainage pathways, Yamamoto et al. (16) proposed vascularized lymph vessel transplantation (VLVT), which is intended to facilitate lymphatic drainage reconstruction while simultaneously providing soft-tissue coverage and may therefore represent a potential strategy for the prevention and treatment of PTL.

However, clinical awareness of PTL after open fractures of the lower leg remains limited. It remains unclear which patients require intensified health education and regular surveillance, which patients may benefit from early identification and complete decongestive therapy (CDT), and whether prophylactic lymphatic reconstruction warrants future evaluation in selected high-risk patients. Therefore, identifying factors associated with PTL and establishing a risk-stratification strategy are important for optimizing postoperative follow-up, early screening, timely intervention, and individualized reconstructive decision-making. Such measures may help limit disease progression and improve long-term limb-salvage outcomes and patient satisfaction. Accordingly, this retrospective cohort study aimed to investigate clinical factors associated with PTL after open fractures of the lower leg and to provide evidence to support the screening of high-risk patients and clinical decision-making.

## 2 Materials and methods

### 2.1 Study design and patient selection

**Figure 2:**
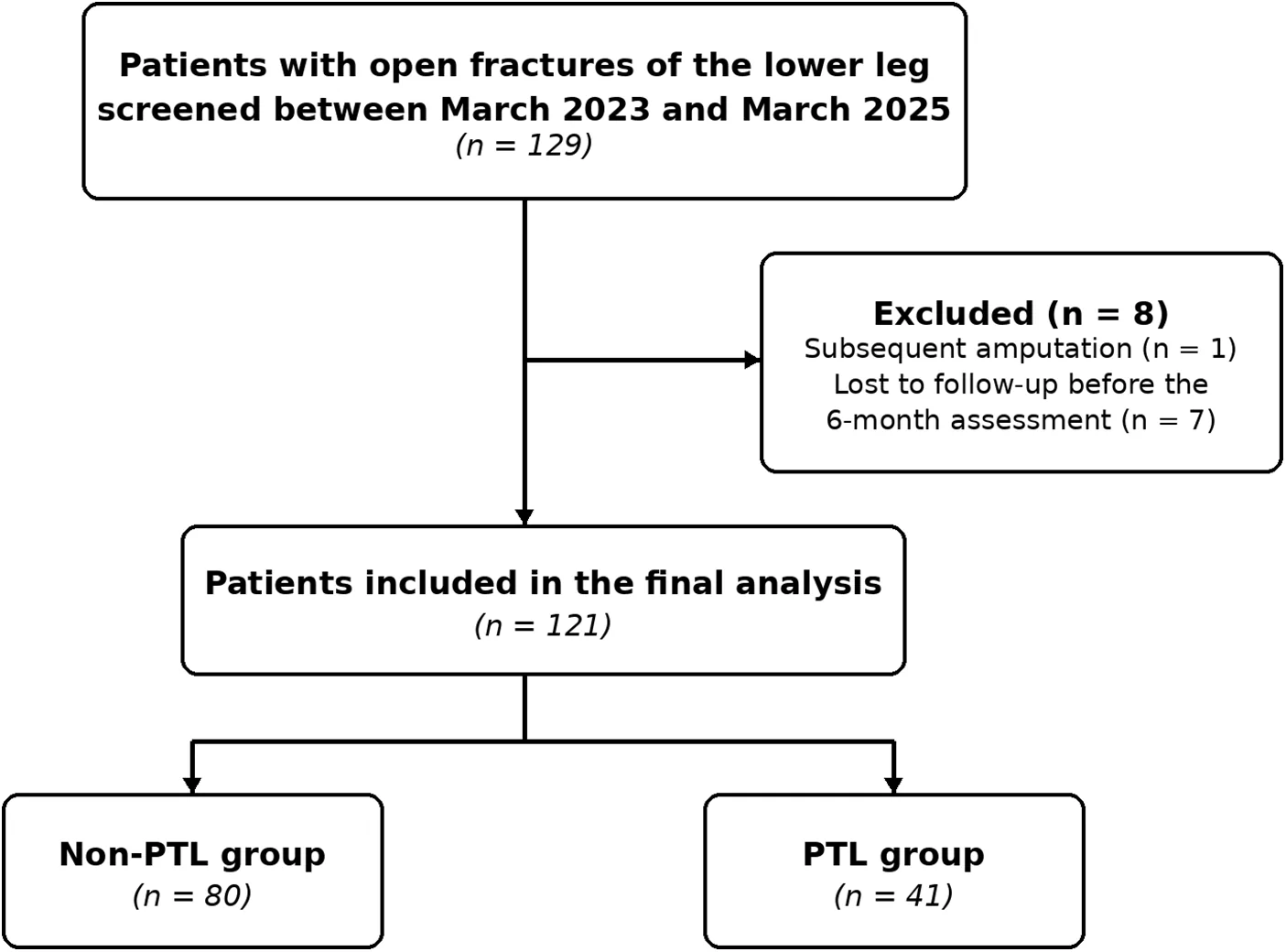
Flow diagram of patient selection according to the Strengthening the Reporting of Observational Studies in Epidemiology (STROBE) guidelines. PTL, post-traumatic lymphedema.

This retrospective cohort study screened patients with open fractures of the lower leg who were treated at Suzhou Ruihua Orthopedic Hospital between March 2023 and March 2025. Eligibility was assessed according to the predefined inclusion and exclusion criteria presented in Table 1. Of the 129 patients screened, eight were excluded: one underwent subsequent amputation and seven did not complete the 6-month follow-up. The final analytic cohort comprised 121 patients. The patient-selection process is shown in Figure 2. The study protocol was approved by the Ethics Committee of Suzhou Ruihua Orthopedic Hospital, and the requirement for written informed consent was waived because of the retrospective study design and the use of anonymized data.

**Table 1:** Eligibility criteria for study participation.

No.	Criterion
INCLUSION CRITERIA	
1	A confirmed Gustilo–Anderson type I–IIIC open fracture of the lower leg treated with limb-salvage surgery
2	Clear consciousness and adequate communication ability
3	Complete clinical data
4	A postoperative follow-up duration of at least 6 months
EXCLUSION CRITERIA	
1	Eventual amputation during follow-up
2	Psychiatric disease or communication disorders preventing reliable assessment
3	A history of lower-leg surgery, radiotherapy, or lymphatic disease before the index injury
4	Comorbidities that could affect lymphatic circulation or limb-edema assessment, including malignancy, congestive heart failure, varicose veins, deep venous thrombosis, thyroid disease, and renal insufficiency

### 2.2 Data collection

Clinical information was extracted from electronic medical records, operative records, and postoperative follow-up records. The variables included (1) baseline characteristics: sex, age, body mass index (BMI), smoking history, hypertension, and diabetes mellitus; (2) injury characteristics: injured side, injury mechanism, Gustilo–Anderson type, wound area, and time from injury to the first debridement procedure; and (3) treatment-related variables and postoperative complications: postoperative infection, hypoproteinemia, and skin and soft-tissue necrosis-related defects. Reconstructive procedures were recorded, and detailed information is provided in Supplementary Table S1.

### 2.3 Outcome definition

The 6-month assessment point was selected with reference to previous research (16) and was applied uniformly to all patients to improve diagnostic consistency, reduce potential assessment bias, and enhance the comparability of the findings. A uniform two-stage diagnostic protocol was applied to all patients. At the 6-month follow-up, all patients underwent clinical screening based on medical history and physical examination, with particular attention to persistent swelling distal to the injured area and a positive Stemmer sign. Patients presenting with both findings subsequently underwent indocyanine green lymphography, venous ultrasonography, and serum biochemical testing.

Post-traumatic lymphedema was diagnosed when indocyanine green lymphography (ICG-L) demonstrated impaired lymphatic drainage with dermal backflow, provided that deep vein thrombosis had been excluded by venous ultrasonography and hypoalbuminemia had been excluded by serum albumin measurement at the time of assessment. In patients with postoperative infection, formal assessment for post-traumatic lymphedema was performed only after the acute infection had been clinically controlled. Pre-existing lymphatic disorders and other systemic conditions capable of causing limb edema were excluded on the basis of the eligibility criteria and a review of the medical records. The final diagnosis was established by experienced microsurgeons through an integrated assessment of clinical symptoms, physical examination findings, laboratory results, and imaging findings.

### 2.4 Variable definitions

The relevant variables were defined as follows. Postoperative infection was defined as an infection involving the injured limb that occurred after the initial operation and before the 6-month assessment for PTL. Infection was considered present when either an infection with clear clinical manifestations and a positive bacterial culture or a skin and soft-tissue infection, such as erysipelas or cellulitis, requiring antimicrobial treatment was documented. Infections occurring at anatomical sites unrelated to the injured limb were not included. Hypoproteinemia was defined as a serum albumin level below 30 g/L within 1 week after surgery. A skin and soft-tissue necrosis-related defect was defined as a full-thickness skin and soft-tissue defect after trauma that required skin grafting or flap reconstruction. Wound area was defined as the skin and soft-tissue defect area requiring coverage before skin grafting or flap reconstruction after debridement or necrosis-related defect formation; it was estimated as the maximum length multiplied by the maximum width and expressed in cm^2^. Time from injury to operation was defined as the interval between injury and the first debridement procedure and was expressed in hours. The Gustilo–Anderson type was dichotomized as type I/II/IIIA versus type IIIB/IIIC for the extended sensitivity model and the alternative injury-severity model.

### 2.5 Statistical analysis

Data were independently entered into Microsoft Excel (Microsoft Corporation, Redmond, WA, USA) by two investigators and cross-checked for accuracy. Statistical analyses were performed using the Statistical Package for the Social Sciences (SPSS), version 29.0 (IBM Corp., Armonk, NY, USA). Continuous variables were first evaluated for normality using the Kolmogorov–Smirnov test. Normally distributed variables were expressed as mean ± standard deviation and compared using the independent-samples t-test. Non-normally distributed variables were expressed as the median and interquartile range [first quartile (Q1), third quartile (Q3)] and compared using the Mann–Whitney U test. Categorical variables were expressed as counts and percentages and compared using the *χ*^2^ test or Fisher's exact test when any expected cell count was < 5.

Binary logistic regression was used for multivariable analysis. Given the limited number of outcome events, variables were not selected solely on the basis of a univariable *P* value < 0.05. Instead, candidate variables were prespecified according to clinical relevance, intervariable relationships, and the study objectives. The main model included age, diabetes mellitus, postoperative infection, and skin and soft-tissue necrosis-related defects. Although hypertension and hypoproteinemia were associated with PTL in the univariable analysis, they were not included in the main model because they may be influenced by age, diabetes mellitus, infection, and overall systemic status and were not considered proximal factors in the pathway of post-traumatic lymphatic drainage impairment.

To assess the robustness of the study findings, an extended sensitivity analysis model was constructed, incorporating age, hypertension, diabetes mellitus, hypoproteinemia, postoperative infection, skin and soft-tissue necrosis-related defects, and the dichotomized Gustilo–Anderson type. Because wound area, Gustilo–Anderson type, and skin and soft-tissue necrosis-related defects all reflect local soft-tissue injury and share overlapping clinical information, the main model included skin and soft-tissue necrosis-related defects because this variable more directly represents the actual extent of tissue loss and the need for wound reconstruction. Furthermore, an alternative injury-severity model was developed to reduce information overlap and potential overadjustment. In this model, the dichotomized Gustilo–Anderson type replaced skin and soft-tissue necrosis-related defects, while all other covariates remained the same as in the main model. Given the limited number of PTL events relative to the number of covariates, the extended sensitivity model was considered exploratory and was used primarily to assess the robustness of the main findings.

The results of the logistic regression analyses were reported as odds ratios (ORs) with 95% confidence intervals (CIs). Model fit was assessed using the Hosmer–Lemeshow goodness-of-fit test. Variable definitions and coding for the regression analyses are presented in Table 2. Multicollinearity among variables included in the multivariable models was evaluated using the variance inflation factor (VIF), with a VIF < 5 considered to indicate no substantial multicollinearity. All tests were two-sided, and *P* < 0.05 was considered statistically significant.

**Table 2:** Variable definitions and coding for the regression analyses.

Variable	Role and type	Original data	Coding
Post-traumatic lymphedema	Dependent variable; binary	No/Yes	No = 0; Yes = 1
Age	Continuous	Actual age, years	Entered as observed (per 1-year increase)
Diabetes mellitus	Binary	No/Yes	No = 0; Yes = 1
Postoperative infection	Binary	No/Yes	No = 0; Yes = 1
Skin and soft-tissue necrosis-related defect	Binary	No/Yes	No = 0; Yes = 1
Hypertension	Binary	No/Yes	No = 0; Yes = 1
Hypoproteinemia	Binary	No/Yes	No = 0; Yes = 1
Gustilo–Anderson classification (dichotomized)	Binary	I/II/IIIA/IIIB/IIIC	I/II/IIIA = 0; IIIB/IIIC = 1

The coding shown reflects the categories used in the regression analyses.

## 3 Results

### 3.1 Baseline characteristics and between-group comparisons

Among the 121 included patients, 41 (33.88%) developed PTL after surgery, whereas 80 (66.12%) did not. In the between-group comparisons, the two groups differed significantly in age, wound area, hypertension, diabetes mellitus, skin and soft-tissue necrosis-related defects, Gustilo–Anderson type, hypoproteinemia, and postoperative infection (all *P* < 0.05). No significant differences were observed in BMI, sex, time from injury to operation, injury mechanism, injured side, or smoking history (all *P* > 0.05) (Table 3). Among the 121 patients, 61 had skin and soft-tissue defects. Of these, 1 patient underwent skin grafting, 59 underwent free-flap reconstruction, and 1 underwent local pedicled-flap reconstruction. A total of 70 free flaps were used in the 59 patients who underwent free-flap reconstruction, including unilateral anterolateral thigh flaps in 47 patients, bilateral anterolateral thigh flaps in 10 patients, a unilateral anterolateral thigh flap combined with a latissimus dorsi flap in 1 patient, and a medial sural artery perforator flap in 1 patient (Supplementary Table S1). Because reconstructive procedures were predominantly based on anterolateral thigh flaps and several alternative categories included only one patient, no formal method-specific comparison of PTL risk was performed. Among the 18 patients with postoperative infection, all had microbiological confirmation, and two also developed erysipelas. The median interval from the initial operation to postoperative infection was 37 days (interquartile range, 12–87 days).

**Table 3:** Baseline characteristics and between-group comparisons according to post-traumatic lymphedema status.

Variable	Overall (*n* = 121)	Non-PTL group (*n* = 80)	PTL group (*n* = 41)	Statistic	*P* value
BMI, mean ± SD	23.92 ± 3.79	24.16 ± 3.95	23.46 ± 3.45	*t* = 0.96	0.340
Age, median (Q1, Q3), years	52.00 (35.00, 60.00)	43.00 (31.50, 55.25)	57.00 (50.00, 65.00)	*Z* = −3.49	<0.001
Wound area, median (Q1, Q3), cm^2^	48.00 (10.00, 216.00)	15.00 (6.75, 106.00)	260.00 (176.00, 341.00)	*Z* = −6.26	<0.001
Time from injury to operation, median (Q1, Q3), h	2.00 (1.00, 3.00)	2.00 (1.00, 3.00)	2.00 (1.00, 3.00)	*Z* = −1.56	0.119
Sex, *n* (%)				*χ*^2^ = 0.25	0.615
Male	85 (70.25)	55 (68.75)	30 (73.17)		
Female	36 (29.75)	25 (31.25)	11 (26.83)		
Hypertension, *n* (%)				*χ*^2^ = 12.19	<0.001
No	100 (82.64)	73 (91.25)	27 (65.85)		
Yes	21 (17.36)	7 (8.75)	14 (34.15)		
Diabetes mellitus, *n* (%)				–	0.001
No	112 (92.56)	79 (98.75)	33 (80.49)		
Yes	9 (7.44)	1 (1.25)	8 (19.51)		
Injury mechanism, *n* (%)				–	0.112
Collision injury	73 (60.33)	48 (60.00)	25 (60.98)		
Cutting injury	4 (3.31)	4 (5.00)	0 (0.00)		
Compression injury	11 (9.09)	6 (7.50)	5 (12.20)		
Struck-by injury	18 (14.88)	9 (11.25)	9 (21.95)		
Fall injury	15 (12.40)	13 (16.25)	2 (4.88)		
Injured side, *n* (%)				*χ*^2^ = 2.40	0.121
Left	65 (53.72)	47 (58.75)	18 (43.90)		
Right	56 (46.28)	33 (41.25)	23 (56.10)		
Skin and soft-tissue necrosis-related defect, *n* (%)				*χ*^2^ = 18.95	<0.001
No	60 (49.59)	51 (63.75)	9 (21.95)		
Yes	61 (50.41)	29 (36.25)	32 (78.05)		
Gustilo-Anderson type, *n* (%)				–	0.003
I	6 (4.96)	5 (6.25)	1 (2.44)		
II	32 (26.45)	27 (33.75)	5 (12.20)		
IIIA	7 (5.79)	7 (8.75)	0 (0.00)		
IIIB	14 (11.57)	9 (11.25)	5 (12.20)		
IIIC	62 (51.24)	32 (40.00)	30 (73.17)		
Smoking history, *n* (%)				*χ*^2^ = 0.01	0.926
No	79 (65.29)	52 (65.00)	27 (65.85)		
Yes	42 (34.71)	28 (35.00)	14 (34.15)		
Hypoproteinemia, *n* (%)				*χ*^2^ = 8.75	0.003
No	64 (52.89)	50 (62.50)	14 (34.15)		
Yes	57 (47.11)	30 (37.50)	27 (65.85)		
Postoperative infection, *n* (%)				*χ*^2^ = 18.19	<0.001
No	103 (85.12)	76 (95.00)	27 (65.85)		
Yes	18 (14.88)	4 (5.00)	14 (34.15)		

Data are presented as mean ± standard deviation, median (Q1, Q3), or *n* (%), as appropriate. *P* values were calculated using the independent-samples t test, Mann–Whitney U test, chi-square test, or Fisher's exact test. A dash in the Statistic column indicates that Fisher's exact test was used.

SD, standard deviation; Q1, first quartile; Q3, third quartile; PTL, post-traumatic lymphedema.

### 3.2 Multivariable logistic regression analyses

The main multivariable model included age, diabetes mellitus, postoperative infection, and skin and soft-tissue necrosis-related defects. Increasing age, postoperative infection, and skin and soft-tissue necrosis-related defects were independently associated with PTL after open fractures of the lower leg (all *P* < 0.05). Each 1-year increase in age was associated with an approximately 5% increase in the odds of PTL (OR = 1.05, 95% CI 1.02–1.09; *P* = 0.002). Patients with postoperative infection had 5.15-fold higher odds of PTL than those without infection (OR = 5.15, 95% CI 1.25–21.19; *P* = 0.023). Patients with a skin and soft-tissue necrosis-related defect had 5.76-fold higher odds of PTL than those without such a defect (OR = 5.76, 95% CI 2.04–16.31; *P* < 0.001). Diabetes mellitus did not reach statistical significance, although the point estimate was elevated (OR = 8.00, 95% CI 0.77–82.97; *P* = 0.081). The Hosmer–Lemeshow test indicated acceptable model fit (*χ*^2^ = 8.03, df = 8; *P* = 0.430) (Table 4).

**Table 4:** Multivariable logistic regression analyses of post-traumatic lymphedema after open fractures of the lower leg.

Variable	Main model OR (95% CI); *P* value	Extended sensitivity model OR (95% CI); *P* value	Alternative injury-severity model OR (95% CI); *P* value
Age, per 1-year increase	1.05 (1.02–1.09); 0.002	1.05 (1.01–1.08); 0.012	1.05 (1.02–1.09); 0.002
Hypertension, yes vs. no	–	3.14 (0.87–11.33); 0.081	–
Diabetes mellitus, yes vs. no	8.00 (0.77–82.97); 0.081	9.54 (0.88–103.82); 0.064	8.28 (0.78–88.29); 0.080
Hypoproteinemia, yes vs. no	–	2.09 (0.75–5.80); 0.156	–
Postoperative infection, yes vs. no	5.15 (1.25–21.19); 0.023	4.91 (1.18–20.39); 0.028	5.74 (1.47–22.50); 0.012
Skin and soft-tissue necrosis-related defect, yes vs. no	5.76 (2.04–16.31); < 0.001	2.87 (0.62–13.22); 0.175	–
Gustilo–Anderson type IIIB/IIIC vs. I/II/IIIA	–	1.94 (0.37–10.22); 0.436	4.78 (1.56–14.62); 0.006

Values are odds ratios (ORs) with 95% confidence intervals (CIs), followed by *P* values. The main model included age, diabetes mellitus, postoperative infection, and skin and soft-tissue necrosis-related defects. The extended sensitivity model additionally included hypertension, hypoproteinemia, and dichotomized Gustilo–Anderson type. In the alternative injury-severity model, dichotomized Gustilo–Anderson type replaced skin and soft-tissue necrosis-related defects. Reference categories were “no” for binary variables and type I/II/IIIA for Gustilo–Anderson type. A dash indicates that the variable was not included in the model.

In the extended sensitivity model, age, hypertension, diabetes mellitus, hypoproteinemia, postoperative infection, skin and soft-tissue necrosis-related defects, and the dichotomized Gustilo–Anderson type were entered simultaneously. Age and postoperative infection remained independently associated with PTL. The effect size of skin and soft-tissue necrosis-related defects decreased and was no longer statistically significant (OR = 2.87, 95% CI 0.62–13.22; *P* = 0.175). Gustilo–Anderson type also showed no independent association (OR = 1.94, 95% CI 0.37–10.22; *P* = 0.436) (Table 4).

In the alternative injury-severity model, increasing age (OR = 1.05, 95% CI 1.02–1.09; *P* = 0.002), postoperative infection (OR = 5.74, 95% CI 1.47–22.50; *P* = 0.012), and Gustilo–Anderson type IIIB/IIIC injury (OR = 4.78, 95% CI 1.56–14.62; *P* = 0.006) were independently associated with PTL. The point estimate for diabetes mellitus remained elevated but did not reach statistical significance (OR = 8.28, 95% CI 0.78–88.29; *P* = 0.080) (Table 4).

No substantial multicollinearity was detected in any of the three models. VIF values ranged from 1.091 to 1.207 in the main model, from 1.162 to 2.528 in the extended sensitivity model, and from 1.095 to 1.167 in the alternative injury-severity model, all of which were below the prespecified threshold of 5 (Table 5).

**Table 5:** Multicollinearity diagnostics for the multivariable models.

Variable	Tolerance	VIF
Main model		
Age	0.917	1.091
Diabetes mellitus	0.873	1.146
Skin and soft-tissue necrosis-related defect	0.854	1.170
Postoperative infection	0.828	1.207
Extended sensitivity model		
Age	0.835	1.198
Diabetes mellitus	0.861	1.162
Skin and soft-tissue necrosis-related defect	0.396	2.528
Postoperative infection	0.817	1.224
Hypertension	0.854	1.171
Gustilo–Anderson classification (binary)	0.443	2.259
Hypoproteinemia	0.844	1.185
Alternative injury-severity model		
Age	0.913	1.095
Diabetes mellitus	0.871	1.149
Postoperative infection	0.857	1.167
Gustilo–Anderson classification (binary)	0.882	1.134

A VIF < 5 was considered to indicate no substantial multicollinearity. All VIF values in the main, extended sensitivity, and alternative injury-severity models were below 5.

VIF, variance inflation factor.

### 3.3 Illustrative case

**Figure 3:**
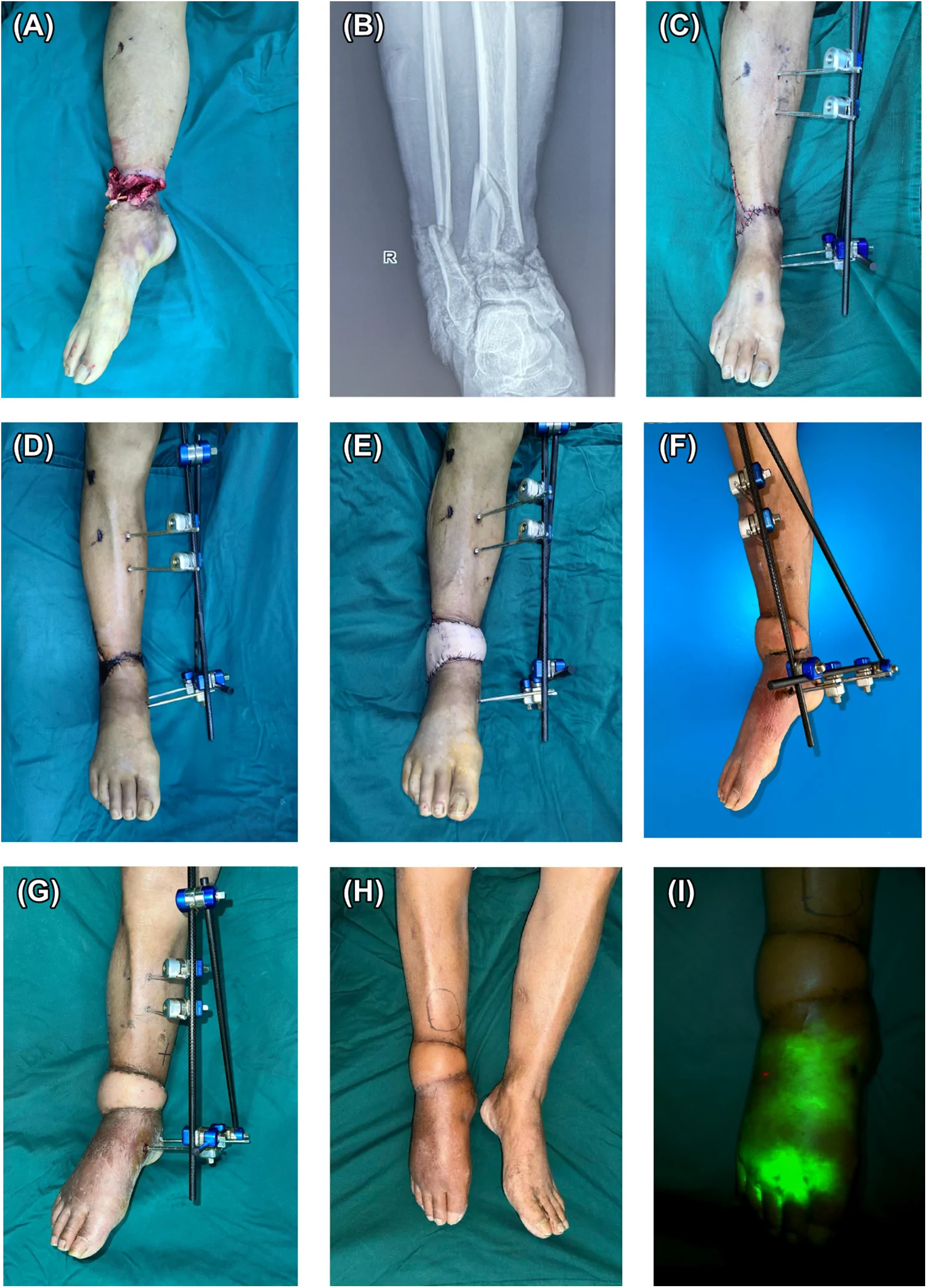
Representative clinical course and diagnostic assessment of post-traumatic lymphedema. **(A)** Wound at admission, showing an incomplete traumatic amputation of the right ankle. **(B)** Anteroposterior radiographs of the tibia and fibula. **(C)** Emergency external fixation with arterial and venous anastomosis. **(D)** Postoperative skin and soft-tissue necrosis-related defect. **(E)** Coverage with a left anterolateral thigh flap. **(F)** Erysipelas distal to the wound at 2 months postoperatively. **(G)** External-fixator pin-tract infection 3 weeks later; bacterial culture of the pin-tract specimen yielded *Klebsiella pneumoniae* subsp. *pneumoniae*. **(H)** Persistent swelling of the injured limb during follow-up. **(I)** Indocyanine green lymphography (ICG-L) showing diffuse dermal backflow.

This case is presented to illustrate the clinical course and diagnostic assessment of PTL. A 70-year-old man with a Gustilo–Anderson type IIIC open tibiofibular fracture underwent vascular repair and anterolateral thigh flap reconstruction. At 2 months postoperatively, the patient developed erysipelas distal to the wound. Three weeks later, an external-fixator pin-tract infection occurred, and bacterial culture of a specimen obtained from the pin tract yielded *Klebsiella pneumoniae* subsp. *pneumoniae*. Persistent swelling of the injured limb was documented during subsequent follow-up. At the 6-month assessment, venous ultrasonography excluded deep vein thrombosis, whereas indocyanine green lymphography demonstrated diffuse dermal backflow consistent with post-traumatic lymphedema (Figure 3).

## 4 Discussion

### 4.1 Skin and soft-tissue injuries

The main model showed that skin and soft-tissue necrosis-related defects were associated with PTL. In the alternative injury-severity model, Gustilo–Anderson type IIIB/IIIC injury was similarly associated with PTL when it replaced the former variable. Both indicators reflect the local injury burden of open fractures but emphasize different aspects: the initial severity of trauma and the actual extent of tissue destruction after injury. The Gustilo–Anderson classification is primarily used to assess initial injury severity, incorporating wound contamination, the extent of soft-tissue injury, and vascular damage. In contrast, skin and soft-tissue necrosis-related defects more directly reflect the actual tissue destruction after trauma and the need for wound reconstruction. Therefore, these two indicators may capture different aspects of the local injury burden associated with PTL.

From an anatomical perspective, the superficial lymphatic vessels of the lower leg are mainly distributed within the skin and subcutaneous tissues. Both skin and soft-tissue necrosis-related defects and Gustilo–Anderson type IIIB/IIIC injuries may therefore involve damage to the superficial lymphatic vessels. Such high-energy injuries are also frequently accompanied by persistent local inflammation. Repeated debridement procedures may cause further lymphatic injury, whereas scar formation may impair lymphatic regeneration and lymphatic drainage (2, 13–15, 17). Yamamoto et al. (16) suggested that spontaneous restoration of lymphatic flow may become limited as the distance between the severed lymphatic ends increases. These mechanisms may partly explain the associations between indicators of severe soft-tissue injury and PTL.

In the extended sensitivity analysis, the associations of Gustilo–Anderson type IIIB/IIIC injury and skin and soft-tissue necrosis-related defects with PTL were attenuated when both variables were included, likely because they capture overlapping aspects of soft-tissue injury. Although no substantial multicollinearity was detected, this model was intended primarily to assess robustness and does not diminish the clinical relevance of either variable. Because reconstruction was predominantly performed using anterolateral thigh flaps, reliable comparisons among reconstructive methods were not possible. Well-designed prospective studies comparing skin grafting, free-flap reconstruction, and local-flap reconstruction are needed to determine how different reconstructive approaches influence lymphatic recovery and the subsequent risk of PTL.

### 4.2 Postoperative infection

Postoperative infection remained independently associated with PTL in both the alternative injury-severity model and the extended sensitivity analysis. Szczesny and Olszewski (4, 5) showed that extravasated blood, bone marrow cells, and bacterial colonization after trauma can sustain local inflammatory activation. Inflammation increases microvascular permeability and tissue-fluid production, whereas inflammatory mediators and bacterial colonization may damage lymphatic endothelium and impair lymphatic contractility and active transport. When tissue-fluid formation exceeds lymphatic compensatory capacity, lymph stasis, tissue fibrosis, and chronic edema may progressively develop (18). Infection and lymphedema may reinforce each other, as recurrent infection can further impair lymphatic drainage and contribute to PTL progression.

However, the temporal relationship between postoperative infection and post-traumatic lymphedema should be interpreted cautiously. In some patients, early post-traumatic edema had not completely resolved before infection developed, and limb swelling persisted after the infection had been controlled. Given the retrospective design, it was not possible to determine whether subclinical lymphatic dysfunction preceded infection and predisposed patients to it, whether infection aggravated pre-existing lymphatic injury, or whether the two processes developed concurrently and interacted with each other. Postoperative infection should therefore be regarded as a factor associated with post-traumatic lymphedema rather than as an established causal determinant.

### 4.3 Age and diabetes mellitus

Increasing age was independently associated with PTL. With aging, lymphatic contractility, valve function, and tissue repair capacity may decline, reducing the ability to re-establish lymphatic drainage after trauma (19–22). Diabetes mellitus showed a high OR but did not reach statistical significance, likely because the number of patients with diabetes mellitus was small and the confidence interval was wide. Hyperglycemia and chronic inflammation related to diabetes mellitus may affect lymphangiogenesis, lymphatic permeability, and lymphatic contractile function (12, 19). Clinically, patients with diabetes mellitus may still be regarded as a potentially high-risk group for PTL and may benefit from more intensive long-term glycemic and wound management after limb salvage, although this interpretation should be made cautiously given the lack of statistical significance and the wide confidence interval (23).

### 4.4 Clinical implications

These findings have practical implications. Postoperative surveillance for PTL should be intensified for older patients and those with postoperative infection, skin and soft-tissue necrosis-related defects, Gustilo–Anderson type IIIB/IIIC injuries, or diabetes mellitus. Particular attention should be given to infection control, wound management, patient education, and persistent limb swelling. Based on the lymph-axiality concept proposed by Yamamoto et al. (16), alignment of lymphatic vessels within the flap with the axial ends of recipient lymphatic vessels may enable physiological restoration of lymphatic flow through natural lymphatic regeneration. When lymphatic axiality is accurately incorporated into flap design and transfer, this strategy may restore lymphatic drainage during soft-tissue reconstruction without requiring additional lymphatic anastomosis (17, 24–26). This strategy warrants prospective evaluation to determine whether it can reduce the incidence of PTL in selected high-risk patients. However, the present study was limited to a risk-factor analysis and did not directly compare this technique with conventional soft-tissue reconstruction; therefore, it provides no direct evidence of preventive efficacy.

### 4.5 Limitations

This study has several limitations. First, its single-center retrospective design, modest sample size, and loss of seven eligible patients to follow-up may have introduced selection bias. Second, only 41 patients developed PTL, and the small number of patients with diabetes mellitus resulted in an imprecise estimate with a wide confidence interval. Residual confounding related to injury severity and reconstructive management may also remain. Third, Gustilo–Anderson type and skin and soft-tissue necrosis-related defects may partly overlap as indicators of injury severity. Fourth, assessment at 6 months may not have completely distinguished prolonged post-traumatic edema from established PTL, and the long-term evolution of PTL could not be evaluated. Finally, the retrospective design did not allow the temporal relationship between lymphatic dysfunction and postoperative infection to be determined.

## 5 Conclusions

Increasing age and postoperative infection were consistently associated with PTL after open fractures of the lower leg. Skin and soft-tissue necrosis-related defects were associated with PTL in the main model, whereas Gustilo–Anderson type IIIB/IIIC injuries were associated with PTL in the alternative model; both associations were attenuated in the extended sensitivity model. Diabetes mellitus was not statistically significant, although a possible association cannot be excluded. These findings support structured postoperative surveillance and warrant confirmation in larger prospective studies with serial lymphatic assessment.

## Data Availability

The original contributions presented in the study are included in the article/Supplementary Material, further inquiries can be directed to the corresponding author.

## Supplementary material

Supplementary Table S1 Distribution of reconstructive procedures among patients with skin and soft-tissue necrosis-related defects.
