# Supplementary material for: Risk factors for post-traumatic lymphedema after open fractures of the lower leg: a retrospective cohort study
Source: Front Surg. 2026 Sep 11;13:1931485. doi: 10.3389/fsurg.2026.1931485 (PMC13614756; doi:10.3389/fsurg.2026.1931485)
Supplement: Supplementary file 1 [file Datasheet1.docx]

**Table S1. Distribution of reconstructive procedures among patients with skin and soft-tissue necrosis-related defects**

| **Reconstructive procedure** | **Overall (n = 61)** | **Non-PTL group (n = 29)** | **PTL group (n = 32)** |
| --- | --- | --- | --- |
| Skin grafting | 1 (1.6) | 1 (3.4) | 0 (0.0) |
| Unilateral anterolateral thigh free flap | 47 (77.0) | 23 (79.3) | 24 (75.0) |
| Bilateral anterolateral thigh free flaps | 10 (16.4) | 3 (10.3) | 7 (21.9) |
| Anterolateral thigh free flap combined with latissimus dorsi free flap | 1 (1.6) | 0 (0.0) | 1 (3.1) |
| Medial sural artery perforator free flap | 1 (1.6) | 1 (3.4) | 0 (0.0) |
| Local pedicled flap | 1 (1.6) | 1 (3.4) | 0 (0.0) |
| **Total** | **61 (100.0)** | **29 (100.0)** | **32 (100.0)** |

Note: Percentages were calculated using patients rather than individual flaps as the denominator. Among the 61 patients with skin and soft-tissue necrosis-related defects, 59 underwent free-flap reconstruction using 70 free flaps, one underwent local pedicled-flap reconstruction, and one underwent skin grafting. Because several reconstructive categories contained only one patient, no formal between-group statistical comparison was performed. PTL, post-traumatic lymphedema.
